# The Impact of Copper Ions on the Activity of Antibiotic Drugs

**DOI:** 10.3390/molecules28135133

**Published:** 2023-06-30

**Authors:** Bojana Božić Cvijan, Jelena Korać Jačić, Milica Bajčetić

**Affiliations:** 1 Department of Pharmacology, Clinical Pharmacology and Toxicology, Faculty of Medicine, University of Belgrade, 11000 Belgrade, Serbia; bojanabozic87@gmail.com; 2 Life Sciences Department, Institute for Multidisciplinary Research, University of Belgrade, Kneza Višeslava 1, 11000 Belgrade, Serbia; jskorac@gmail.com; 3 Clinical Pharmacology Unit, University Children’s Hospital, 11000 Belgrade, Serbia

**Keywords:** antibiotics, copper, antimicrobial activity, resistance

## Abstract

Copper (Cu) is an essential trace metal and its concentration in body plasma is tightly regulated. An increase in Cu concentration in body fluids is observed in numerous pathological conditions, including infections caused by microorganisms. Evidence shows that Cu ions can impact the activity of antibiotics by increasing efficiency or diminishing/neutralizing antibiotic activity, forming complexes which may lead to antibiotic structure degradation. Herein, we represent the evidence available on Cu–antibiotic interactions and their possible impact on antimicrobial therapy efficiency. So far, in vitro studies described interactions between Cu ions and the majority of antibiotics in clinical use: penicillins, cephalosporins, carbapenems, macrolides, aminoglycosides, tetracyclines, fluoroquinolones, isoniazid, metronidazole. In vitro-described degradation or lower antimicrobial activity of amoxicillin, ampicillin, cefaclor, ceftriaxone, and meropenem in the presence of Cu ions suggest caution when using prescribed antibiotics in patients with altered Cu levels. On the other hand, several Cu-dependent compounds with antibacterial activity including the drug-resistant bacteria were discovered, such as thiosemicarbazones, disulfiram, dithiocarbamates, 8-hydroxiquinoline, phenanthrolines, pyrithione. Having in mind that the development of new antibiotics is already marked as inadequate and does not meet global needs, the potential of Cu–antibiotic interactions to change the efficiency of antimicrobial therapy requires further investigation.

## 1. Introduction

The potential impact of copper ions on antibiotic activity has been pointed out recently [1]. Evidence shows that Cu can impact the activity of antibiotics in a dual way: (1) by increasing the antimicrobial effects, as described in Cu-dependent compounds, or (2) through coordination and/or redox interactions [2,3,4,5]. Copper ions-dependent compounds with antibacterial activity have been identified against several multi-resistant bacteria [1]. These findings are important for further combat with bacterial antimicrobial resistance (AMR), especially if we know that in 2019, the estimated mortality associated with AMR was 5 million, including 1.27 million deaths directly attributable to resistance [3]. Additionally, opposite to the rising trend of bacterial resistance is the speed of antibiotic discovery [2]. The ineluctable conclusion is the urge for a different approach in antibiotic discovery and synthesis [1,2]. Ideally, studies in the future will provide Cu-dependent compounds as a new entity of antimicrobials, effective by themselves and with the ability to restore the activity of current antibiotics by reversing antibiotic resistance [4]. The other side of the medal is the possibility for Cu to interact with antibiotics. Having in mind the role of copper in the native immune system and the fact that its concentration increases during infectious diseases, the possibility to affect the activity of antibiotics through complexation and redox interactions became a focus [1,5]. The purpose of this review is to discuss the dual impact of Cu ions on antibiotic activity currently proposed in the literature and their potential applications in clinical practice.

## 2. Copper’s Role in the Human Organism

Cu is a transition metal with the capacity to form 4–6 coordinative bonds and in serum, exists as cuprous (Cu^+^) and cupric (Cu^2+^) cations [5,6]. As an essential trace metal, Cu plays a crucial role as a cofactor in numerous enzymatic reactions catalyzed by cytochrome c oxidase, dopamine ß hydroxylase, tyrosinase, lysyl oxydase, and Cu-dependent superoxide dismutase [6,7,8]. Due to the roles of the mentioned enzymes in the human organism, it is clear that copper ions have a great impact on electron transfer during cellular respiration, energy generation, iron oxidation, pigment and connective tissue formation, neurotransmitter and antioxidant biosynthesis [9,10,11,12,13,14].

Copper ion concentration is tightly regulated, and any imbalance may lead to a number of pathological conditions [7]. Deficiency, as well as excess in Cu concentration, is described in several pathological conditions, such as tuberculosis, diabetes mellitus, prion diseases, Wilson’s disease, Menkes disease, cancer, anemia, atherosclerosis, arrhythmias, Alzheimer’s disease, Parkinson’s disease, dyslipidemia, obesity, non-alcoholic fatty liver disease, osteoporosis, depigmentation, lymphosarcoma, hypotonus, apnea syndrome, infections, inflammation [5,7,15,16]. In addition to all the mentioned diseases, Cu ions imbalance in the human body during pregnancy can have an impact on fetus development [17]. Cu deficiency in utero may lead to abnormalities of cardiovascular, skeletal, neurological, and immunological systems [17,18]. Opposite this, increased Cu levels are observed in full-term and premature infants with or without hemolytic jaundice [9]. After the discovery that Cu ions have an important role as components of the innate immune system but also as agents that may cause cytotoxic effects, interest in this ion increased [1,19]. The study from 2000 on isolated hepatocytes explained cytotoxicity induced by Cu ions in detail. It was shown that the incubation of hepatocytes with Cu ions rapidly increases the production of free radicals [19]. Having in mind all the roles of copper ions in the human body, as well all the mentioned pathological conditions linked with its imbalance in the human organism, it is not surprising that the possible antimicrobial, antiviral, anti-inflammatory, and antitumor potential of copper ion metal chelators is being thoroughly studied [7,20].

### Copper Pathway in Human Organism

**Figure 1:**
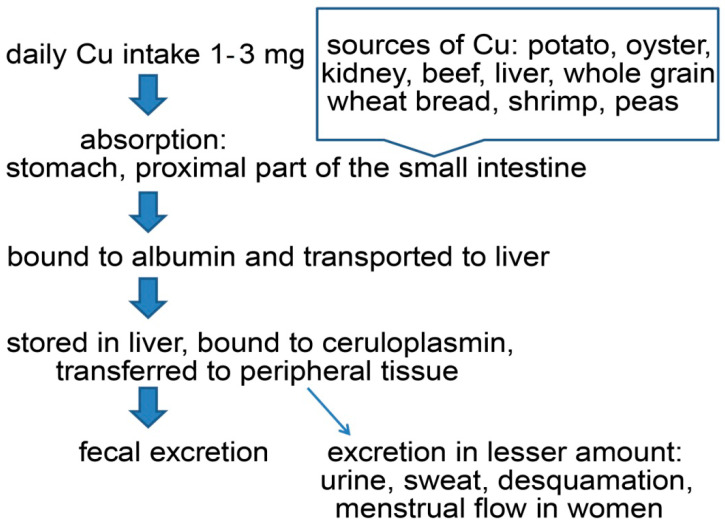
Cu pathway in human organisms.

Cu is an essential trace element in both humans and animals [17]. The total concentration of Cu in the adult human body is around 100 mg (1.6–2.4 mg/kg) [6,17]. Plasma Cu levels in one-month-old neonates are significantly lower compared to adults, achieving a maximum value at 2-to-5 years of age, then decreasing gradually [21]. Depending on the country, differences regarding the recommended daily allowance of copper in the human population can be observed [17]. The usual daily copper intake is one to three mg [15]. Factors such as age, gender, type of food, amount of dietary Cu, and oral contraceptives can lead to a variation in absorption rate from 12% up to 71% [17]. Food such as potato, oyster, kidney, beef, liver, whole grain wheat bread, shrimp, and peas are great sources of copper [17] (Figure 1).

After food ingestion, copper absorption occurs mainly in the stomach and proximal part of the small intestine [7,17]. By binding to Cu transporter 1 (CTR1), copper ions enter the enterocytes, and by binding to Cu-transporting ATPase 7 (ATP7A), they enter the bloodstream [15]. ATP7A provides Cu ions for the enzymes that need copper during its synthesis [15]. Absorbed copper is bound to albumins and transported to the liver. Hepatocytes have a crucial role in copper homeostasis and even half of the total Cu concentration is stored in the liver [21]. Bound to ceruloplasmin, and to albumin at a lesser extent, copper is transferred to peripheral tissue [7]. In cases of copper excess, changes in both absorption rate and biliary excretion are observed [17]. Cu is excreted into the bile duct through ATP7B [9]. Urinary copper excretion is considerably low, while other routes of copper excretion such as sweat, desquamation, and menstrual flow in women are less significant [17].

On the other hand, inorganic copper from drinking water or copper supplements bypasses the liver in a higher percentage. In this way, an increased copper pool in the blood may be achieved. The described mechanism has possible clinical implications due to the possibility of copper penetrating the blood–brain barrier [7].

## 3. Copper as an Antimicrobial Agent

The oldest medical application of Cu was over two thousand years before Christ, in Egypt, where copper was used as an agent to sterilize wounds and water [22]. This practice was later continued in Persia, Greece, and Rome [23]. The use of Cu for medical purposes was at its peak during the 19th and 20th centuries, when Cu was used to treat chronic adenitis, eczema, impetigo, tubercular infections, lupus, syphilis, anemia, chorea, and facial neuralgia [22]. The discovery of commercially used antibiotics put copper’s antimicrobial role in the second perspective [22]. Over the years, the widespread use of antibiotics has led to bacterial resistance [19,22] and once again, focus was on possible alternative approaches such as copper complexes. The use of Cu on various hospital surfaces proved to be very beneficial: the presence of copper compounds on doors, doorknobs, and stethoscopes in hospitals has led to a reduction of the number of bacteria [22,23]. Cu surfaces kill microorganisms in a process named contact killing, which occurs on a timescale of minutes to hours [23]. Studies have shown that although the administration of free Cu is neither justified nor rational, the use of small ligand complexes may have potential benefits [1,24,25]. Synthesis and use of nanoparticles (particularly, those made of copper and silver) were shown to be a great tool for preventing bacterial and fungal activity by contact killing and by degradation of bacteria DNA plasmid, preventing the transfer of resistance between microorganisms [24,25]. It is hypothesized that the release of ions is a main contributor to the antimicrobial properties of nanoparticles [23,24]. So far, nanoparticles showed high antimicrobial activity. In the laboratory settings, the higher copper content of alloys, higher temperature, and relative humidity increase the efficacy of contact killing [22]. Due to the very fast and complete degradation of plasmid DNA by contact killing, so far, no bacteria completely resistant to Cu contact killing have been discovered [22].

### The Effects of Elevated Copper on Bacteria

Nowadays, there is a growing interest in Cu complexes due to their possible role in preventing or even reversing the antimicrobial resistance to classic antibiotics [4]. So far, it seems that translation studies from in vitro to in vivo are lacking. Such observations could be explained by a lack of information on the molecular mechanisms underlying Cu–antibiotic interactions at physiological conditions.

The precise mechanism of Cu-induced bacterial damage is not fully explained. Several possible mechanisms were proposed. One of the explanations for metal toxicity is the ability of cupric and cuprous ions to form reactive oxygen species (ROS) [26]. Studies have shown that after bacteria and yeast were exposed to Cu, genes involved in ROS elimination are upregulated, indirectly implying that ROS plays a crucial role in Cu-mediated cellular damage [23,27]. In the presence of superoxide or reducing agents, Cu^2+^ can be reduced to Cu^+^ which is able to form hydroxyl radicals (OH^•^) from hydrogen peroxide via the Fenton-like reaction [1,26].

O_2_¯ + Cu^2+^ → O_2_ + Cu ^+^

Cu ^+^ + H_2_O_2_ → Cu^2+^ + OH¯ + OH^•^

The hydroxyl radical is the most powerful oxidizing radical, capable of reacting with practically every biological molecule, and it initiates oxidative damage and consequently leads to cellular death [26,28].

Hard–soft acid-base theory (HSAB theory or Pearson’s acid-base theory) classifies transition metals according to their preferences for specific ligands. Soft acids such as Cu^+^ and borderline acids such as Cu^2+^ tend to tightly bind with soft bases such as sulfhydryl groups (R-SH). The antibacterial activity of metal ions is proportional to their affinity for binding to the sulfhydryl group [23]. Cu covalent bonding to thiol leads to protein disulfides and depletion of antioxidant reserves [23,29]. Depending on the characteristics of each sulfhydryl group, the result can be complex formation or redox reactions [22].

2 Cu^2+^ + 2RSH → 2 Cu^+^ + RSSR +2H^+^

2 Cu^+^ + 2H^+^ + O_2_ → 2 Cu^2+^ + H_2_O_2_

Hydroxyl radicals produced from hydrogen peroxide in Fenton reaction lead to cell damage. Possible mechanisms of Cu toxicity are also competition with other metal ions for binding with proteins and Cu displacement of iron from iron–sulfur clusters in an oxygen-independent way as well as the formation of coordinative compounds with organic and inorganic ligands [30,31].

## 4. Copper-Dependent Compounds

Several Cu-dependent compounds have potential therapeutic applications. The examples of Cu-dependent compounds are presented in Table 1. The main proposed mechanisms of action of copper-dependent compounds will be discussed briefly in the following section.

### Mechanism of Cu-Dependent Compounds

So far, several hypotheses regarding Cu-dependent compounds have been studied. In the case of disulfiram, a “Trojan horse” model has been proposed. The first step is Cu^2+^-mediated reduction of disulfiram and the creation of diethyldithiocarbamate (DETC, L1) which later chelate with Cu^2+^ ions, [Cu(L1)_2_] [1,34]. A shield formed in this way can provide Cu ions to easily overcome bacterial defense mechanisms [1,34]. Consequently, more labile Cu is inside the bacterial cell, which leads to ROS production, metal cofactor replacement, and impact on iron–sulfur clusters [34].

In the mechanism of action of glyoxal-bis(N_4_-methylthiosemicarbazonato) (L2), another example of Cu-dependent compounds, [CuL2], has been described in detail against N. gonorrhoeae, whose Cu detoxification system is underdeveloped compared to most of the other bacteria [32]. The first step is the inability of a bacteria efflux pump to combat Cu overload. Once inside the bacteria cell, Cu ions influence NADH dehydrogenases and initiate a redox cycle inside N. gonorrhoeae. For the first time, this work considered Cu^2+^ action as target specific [33,39]. Since no effect on normal microflora has been observed, this example can serve as an opportunity to repurpose already approved drugs for new medical uses and possibly lead to a promising way of combating bacteria resistance [32,39].

Detailed analysis showed that 8-hydroxyquinoline acts as a Cu^2+^ ionophore. The presence of other metals such as Zn, Fe, and Mn has no activity on the Cu-8-hydroxyquinoline (L3) complex, [CuL3], which kills *M. tuberculosis* selectively within infected macrophages [35].

It is suggested that pyrithione acts as a copper ionophore, enabling it to enter cells and distribute across intracellular membranes. Copper–pyrithione complex leads to growth inhibition of the fungus *Malassezia globosa* (*M. globosa*) [40]. Several years later, another study pointed to a complex formation between copper and pyrithione. Adding the copper–pyrithione complex to amikacin led to growth inhibition of an amikacin-resistant *K. pneumoniae* [38].

Neocuproine (L4), a well- known copper complexing compound, exhibited intracellular activity. Under copper-activated conditions, the mentioned complex, [Cu(L4)_2_], was highly effective against MRSA [33].

Other promising copper-dependent agents are pyrazolopyrimidinones. Copper- 5-benzyl-3-(4-chlorophenyl)-2-methyl-4H,7H-pyrazolo[1,5-a]pyrimidin-7-one (L5) complexes, [Cu(L5)_2_], showed a significant impact on *S. aureus* through several mechanisms: depletion of cellular ATP, electrolyte imbalance, and inability to control the influx of protons, while the cell membrane remained intact [37].

Complex Cu-1,10 phentaroline (phen) possessed high antibacterial activity against metronidazole-resistant *Trichomonas vaginalis* (*T. vaginalis*), dematiaceous fungus *Phialophora verrucosa* (*P. verrucosa*), clinically relevant yeast *Candida albicans* (*C. albicans*), multidrug-resistant strains of *Candida haemulonii* species complex, flamentous fungus *Scedosporium apiospermum* (*S. apiospermum*), *Saccharomyces cerevisiae* (*S. cerevisiae*), *E. coli*, MRSA, carbapenemase-producing *A. baumannii*, and multidrug-resistant *P. aeruginosa*. As a possible explanation, the overtone concept was proposed. On chelation, the polarity of copper ion is reduced, which favors its permeation through the lipid barriers allowing an impact on bacteria [41,42]. Furthermore, the potential of phentaroline–copper complex was described in in vivo studies. The complex possessed a non-mutagenic profile and low toxicity in laboratory settings on a mice model [42,43].

A recently published study has added a new compound to the growing list of copper-dependent drug classes. Authors described an adamantyl-bearing pyrazolyl–thioureas (APT)-6K/ampicillin synergy with an improved safety profile compared to other copper-dependent compounds. Another major breakthrough is the described ability of the mentioned compound to reverse drug resistance against clinically relevant antibiotics, such as ampicillin [4]. So far, it has been hypothesized that copper-dependent compounds can affect different ATP-generating processes, such as oxidative phosphorylation and glycolysis. Decreased ATP levels may restore antibiotic sensitivity in several multi-drug resistant bacteria: *S. aureus* to polymyxins with oligomycin; *M. tuberculosis* to β-lactam antibiotics with 2-amino imidazoles [4].

## 5. Copper Interactions with Antibiotics

Generally, antibiotic interactions can be classified as synergistic or antagonistic, depending on whether the drug combination leads to increased or decreased antibiotic activity [44]. Furthermore, by decreasing antimicrobial activity, interactions may be involved in the underlying mechanisms of bacterial resistance [5]. Interactions between antibiotics and metal ions can have various consequences, implying the need to understand and investigate each interaction separately. Searching literature, we gained insight into numerous studies that described antibiotic–metal interactions with various effects. An additional aggravating factor is a possible discrepancy between obtained in vitro results compared to in vivo results. Therefore, knowledge based on well-designed in vitro and in vivo studies investigating which antibiotic–metal interaction may lead to decreased or more efficient antimicrobial therapy are essential for the selection of the best available therapy.

### 5.1. Copper Interactions with Penicillins and Cephalosporins

Special attention should be paid to Cu^2+^ interactions with one of the most frequently prescribed antibiotic classes, β-lactam antibiotics. β-lactam antibiotics are bactericidal drugs, containing the β-lactam ring in their chemical structure [45]. The β-lactam ring, 6-aminopenicilloic acid, is the key to the synthesis and modification of penicillins. They are classified into penicillins, cephalosporins, carbapenems, and monobactams [45]. In penicillins, cephalosporins, and carbapenems, the β-lactam ring is fused to a 5- or 6-member ring; in monobactams, the β-lactam ring is monocyclic [46]. The β-lactam ring is mainly responsible for the drug mechanism of action: the ability to block the bacterial cell wall synthesis as a result of their covalent binding to penicillin-binding proteins (essential enzymes in the synthesis of peptidoglycan) [46].

Penicillins are natural or synthetic antibiotics derived from fungi, with molecular formula R-C_9_H_11_N_2_O_4_S. All penicillins contain three basic components: a thiazolidine ring, a β-lactam ring, and a side chain [47]. Penicillin V was synthesized by acylation of 6-aminopenicilloic acid, while aminopenicillins, ampicillin, and amoxicillin were formed, adding an amino group to the penicillin structure [48]. In parallel with the development of semi-synthetic compounds was the isolation of cephalosporin C from the Cephalosporium acremonium strain [49]. The main difference between cephalosporins and penicillins is the presence of 7-aminocephalosporinic acid instead of 6-aminopenicilloic acid [48].

During the years, using different analytical techniques, the interactions between β-lactam antibiotics and metal ions, among them Cu, Ag, and Zn, were studied [50]. First studies regarding β-lactam complexes with Cu^2+^ were conducted at pH values different from physiological conditions [51,52] and although obtained results were hard to interpret, it was implied that degradation of penicillin V and penicillin G occurred [52]. Under mild acidic conditions, both penicillin G and V were hydrolysed by the cupric ion into penicilloic acids [52]. Over fifty years later, the first study that described copper and β-lactam interactions under a physiological set-up was published [5].

Using UV-VIS spectrophotometry and electron paramagnetic resonance (EPR), a study published in 2018 showed that penicillin G does not form a complex with Cu^2+^ under physiological conditions, but a certain degree of slow penicillin G degradation could not be excluded [5]. Interesting data regarding antimicrobial activity were described in the same study: in the presence of copper ions, penicillin G showed decreased activity against *S. aureus* [5]. Since no interactions between Cu and penicillin were observed, changed antimicrobial activity was explained by a possible slow copper-induced degradation due to the time and temperature required for minimum inhibitory concentration (MIC) assay to conduct [5].

A study carried out in non-physiological (pH values lower compared to physiological values) conditions described Cu^2+^-mediated hydrolysis of ampicillin, amoxicillin, and cephalexin and the possible reduction of the antimicrobial effects [53]. Later on, possible coordination sites of β-lactams to Cu^2+^ were suggested: β-lactam nitrogen, carboxylate group, carbonyl group, and side-chain amide nitrogen [54,55]. Using spectrophotometry, EPR spectroscopy, and electrochemical methods, one of the latest published studies proposed a primary amine group on the side chain and nitrogen of the amide group as possible donor atoms forming a coordinative bond between Cu^2+^ and amoxicillin (L6) [CuL6], ampicillin (L7) [CuL7], and cephalexin (L8) [CuL8] [5]. Mentioned methods also provided proof that these antibiotics may increase the Cu pool in body fluids [5]. Having in mind all the roles that labile Cu plays in the organism, the information of its increased concentration can be of great clinical value.

The impact of Cu-β lactam antibiotics interactions on bacterial susceptibility is still very complicated and results are inconsistent. On the one hand, a study from 2010 showed improvement in the antibacterial activity of amoxicillin and ampicillin after binding with Cu^2+^ against *B. subtilis* and *E. coli* due to a change in particle size [56]. On the other hand, a recently published study showed decreased activity of ampicillin and amoxicillin after complexation with Cu^2+^ against *E. coli* and no change in the activity of ampicillin–Cu and decreased amoxicillin–Cu activity against *S. aureus* [5]. 

Over the years, several studies described Cu–cephalosporin interactions and their antimicrobial potential [5,55,57,58,59,60,61,62]. Possible explanations for enhanced antimicrobial activity of Cu–antibiotic complexes, compared to antibiotic alone, are the reduced polarity and increased lipophilic nature of the central metal ion environment, and more efficient diffusion, which altogether cause the complex to enter bacterial cells leading to increased bioavailability and activity of the drug [55,63]. This explanation was applied to a Cu complex with cefazolin, cephalexin, etc. [55,64]. The antimicrobial activity of Cu–cephalosporin complexes varies widely depending on used cephalosporin and tested microorganisms (Table 2).

Interesting results were observed in the view of Cu^2+^ interactions with cefaclor, cephalosporin of the second generation. In the presence of Cu ions, degradation of cefaclor has been described [5]. Using oximetry, significant oxygen consumption was detected, providing insight into the antibiotic degradation kinetics [5]. Regarding antimicrobial activity, cefaclor showed slightly increased activity toward *E. coli*, which can be explained by the production of hydrogen peroxide [5]. The seemingly contradictory result of improved antimicrobial activity on the one side, and described degradation of cefaclor on the other, would probably be easily explained in in vivo study when catalase would rapidly remove hydrogen peroxide. So far, it is clear that given the diversity of the nature of Cu–antibiotic complexes, each compound needs to be analyzed separately and conclusions cannot be generalized [33]. Only when the precise mechanism of Cu–antibiotic complexation is fully described and when coordination sites of antibiotics bound to copper ions are recognized can the true extent of the interaction be understood and explained. Furthermore, the possible effects of the complex in clinical settings should be interpreted only at pH-relevant values. We can observe that data regarding interactions of the newest cephalosporins with copper ions are missing.

### 5.2. Copper Interactions with Carbapenems

Carbapenems are β-lactam antibiotics with broad-spectrum activity [65]. They represent the first line of treatment for infections caused by the most resistant bacteria, among which are dominant bacteria from the ESKAPE group (*E. faecium*, *S. aureus*, *K. pneumoniae*, *A. baumannii*, *P. aeruginosa*, Enterobacter species) [66]. Carbapenems have a penicillin-like five-membered ring, but the sulfur at C-1 in the five-membered ring is replaced by a carbon atom and a double bond between C-2 and C-3 [66]. Described changes in chemical structure were responsible for their insensitivity to the effects of β-lactamases–carbapenemases [66].

Although data regarding Cu–carbapenem complexes are limited, a recently published study showed that Cu can indirectly improve carbapenem action inactivating B metallo–β-lactamases responsible for the hydrolysis of carbapenems, and consequently decrease the risk of bacterial resistance [65]. Having in mind that the production of carbapenemases is the major mechanism underlying carbapenem resistance [66], the described result can present a valid marker for future investigation trends.

A study published in 2018 described meropenem degradation in the presence of copper ions [5]. Degradation was not followed by oxygen consumption, implying that meropenem does not undergo oxidation in the presence of Cu ions, or that it may even stabilize Cu^+^ ions [5]. Further experiments suggested irreversible oxidation of the sulfide group of meropenem in its copper complex [5]. In line with the described degradation of meropenem in the presence of Cu ions is a drastic decrease in the activity of meropenem against *E. coli* and *S. aureus* [5].

### 5.3. Copper Interactions with Tetracyclines

Tetracyclines are a commonly used antibiotic group in human and veterinary medicine [67]. Their mechanism of action relies on the inhibition of bacterial protein synthesis by preventing the association of aminoacyl tRNA with the bacterial ribosome [68]. In order to reach their target and to pass through membranes, tetracyclines have to be positively charged, most likely forming a complex with magnesium ions [67,68,69]. They contain several important functional groups: five hydroxyl, two carbonyl, and one amide [67]. So far, tetracyclines are known as potential chelating agents, and complexes with different metal ions such as sodium, potassium, magnesium, calcium, cadmium, and lead are described [67].

Since tetracycline molecule possesses several ionizable functional groups, their ability to bind Cu^2+^ is not a surprise [67]. So far, the in vitro model showed the formation of the Cu–tetracycline(L9) complex, [CuL9], and possibility of a tetracycline molecule to act as a Cu sink. According to this in vitro model, the formation of the Cu–tetracycline complex reduces the toxicity of Cu^2+^ and tetracycline [70]. Still, the precise role of this interaction on antibacterial activity is not fully understood [1,69].

### 5.4. Copper Interactions with Fluoroquinolones

Fluoroquinolones are frequently prescribed broad-spectrum antibiotics [71]. Their mechanism of action relies on the inhibition of the enzymes responsible for DNA replication—topoisomerase II (DNA gyrase) and topoisomerase IV [71]. Due to the limited activity of the first discovered quinolones such as nalidixic acid, structural changes to the basic nucleus were introduced. The introduction of a fluorine atom at position 6 of the basic quinolone ring gave rise to a fluoroquinolone with a much-broadened antimicrobial spectrum [71,72]. Numerous studies regarding interactions between fluoroquinolones and metal cations such as Zn, Ni, and Co have been reported [73]. Interactions between fluoroquinolones and Cu^2+^ are also studied. Beside no obvious change in the activity of fluoroquinolones with Cu^2+^ ions on *S. aureus* and increased activity against *E. coli*, an important contribution of these interactions is reported regarding the influx of the Cu–fluoroquinolone complex [1]. The described benefit of complex Cu–levofloxacin(L10)–phenanthroline, [CuL10Phen], is penetration through the outer membrane of Gram-negative bacteria via a non-porin-dependent pathway [72]. Having in mind that porin deficiency is one of the most common causes of fluoroquinolones resistance, an alternative pathway can be of great clinical value [69]. Another possible clinical benefit of the Cu–fluoroquinolones complex can be improved photostability with possibly fewer side effects, as it was shown in the study which described Cu^2+^ interactions with lomefloxacin, (L11), [CuL11phen] [71,74] and sparfloxacin, (L12), [CuL12phenCl] [75]. Increased potency is observed for the Cu–gatifloxacin(L13) complex, [CuL13phenCl]·5H_2_O, compared to an antibiotic alone due to the chelate effect and nature of the ligand [76]. Due to the described potential benefit, copper–fluoroquinolone–phenanthroline complexes are representative of suitable candidates for further metalloantibiotic testing, especially in multi-resistant strains [71,72].

### 5.5. Copper Interactions with Aminoglycosides

Aminoglycosides are useful mainly against aerobic Gram-negative microorganisms, commonly prescribed in combination with other agents [77]. They are bactericidal agents and irreversible inhibitors of protein synthesis [78]. Most clinically used aminoglycosides have an aminocyclitol ring, usually 2-deoxystreptamine, to which various amino sugars are bound at positions 4 and 5, or 4 and 6 [79]. An important obstacle in aminoglycoside use is their side effects [77]. With the aim of overcoming this obstacle, different approaches were investigated.

The role of Cu-aminoglycoside complexes on antimicrobial activity and nephrotoxicity and/or ototoxicity is still controversial [1,2]. On the one hand, there is an explanation that by binding Cu to aminoglycosides, ROS are forming and contribute to aminoglycosides toxicity [80]. Similarly, the coadministration of transition metal chelators and free radical scavengers in animals suppressed aminoglycoside-induced ototoxicity [81]. On the other hand, reports suggest that Cu–aminoglycoside complexes are not forming in vivo because Cu ions are mostly bound to proteins (albumin, ceruloplasmin), and aminoglycosides have a lower binding affinity compared to other chelators [1,78]. It is suggested that the copper–aminoglycoside complex plays a minor role in the toxicity of aminoglycosides due to the fact that complexes cannot withstand the presence of histidine [78].

Regarding antimicrobial characteristics of formed complexes, a study from 1998 showed no changes in the activity of kanamycin A (L14) alone compared to Cu–kanamycin A, [CuL14] [82]. Cu interactions with streptomycin and neomycin are also discussed [69,83]. Detailed analysis showed that complex Cu–neomycin (L15), [CuL15(H_2_0)_4_], has no antimicrobial activity against strains on which neomycin is biologically active such as *E. coli*, *P. aeruginosa*, *Bacillus cereus* (*B. cereus*), *Bacillus subtilis* (*B. subtilis*), *S. aureus*, *S. cervisiae* [83]. As was expected due to streptomycin’s tendency to hydrolyze in the presence of metal ions, copper ions may interfere with its activity [69,80]. Cu binding to capreomycin showed increased efficacy against *M. tuberculosis* [69].

### 5.6. Copper Interactions with Other Antibiotics and Antibiotic Groups

Metronidazole (1-(2-Hydroxyethyl)-2-methyl-5-nitroimidazole) is prescribed for various bacterial and protozoal infections [84]. Since metronidazole contains three types of potential donor atoms, the nitrogen atom of an imidazole ring and oxygen atoms originating from alcohol and nitro groups, the described interactions with Co, Zn, Ru, Pd, and Ag were not a surprise [85]. Nearly 40 years ago, direct interactions were also presented for Cu and metronidazole (L16) [85]. Cu^2+^ binds through imidazole nitrogen and the forms complex [Cu(L16)_2_Cl(H_2_0)]_2_Cl_2_, with higher stability, protected from inactivation by numerous enzymes [86]. A study published in 2022 pointed out that copper complexes with metronidazole or metronidazole benzoate have overall better biological activity compared to each ligand used alone [87].

Vancomycin, a glycopeptide antibiotic, is one of the “last-line” classes of antibiotics, used in the treatment of life-threatening infections caused by Gram-positive bacteria [88]. Interactions between Cu and three nitrogen atoms of vancomycin (L17), [CuL17], as well as high affinity of vancomycin for Cu ions, were described [89]. It is suggested that copper ions can affect the action of vancomycin, especially when vancomycin is used in higher doses [89].

Researchers have observed Cu^2+^ interactions with isoniazid [90], chloramphenicol [91], novobiocin [69], and lincomycin [92]. Interesting data were obtained regarding Cu^2+^ interactions with macrolides. The assumption was that potential differences in stability and structures of Cu–macrolides complexes may define its antimicrobial activity: the higher the formation constant is with Cu^2+^, the more powerful the antibiotic is [93].

## 6. The Effects of Copper on Bacterial Resistance

So far, several factors that may lead to reduced efficiency of antibiotics are recognized. As previously discussed, one of the factors can be Cu’s ability to form complexes with antibiotics which may lead to antibiotic structure degradation. In a wider perspective, as with antibiotics, metals are a source of stress for bacteria and lead to adaptive and protective responses [69]. The presence of Cu^2+^ in the soil even in low concentrations is positively correlated with higher expression of antibiotic-resistant genes [94]. It can be assumed that cross-adaptation, exposure, and adaptation to one stress can lead to better adaptation to another type of stress that may arise later. After feeding animals with food containing Cu additives, resistant strains of Salmonella were registered. Due to the possible transmission of resistant bacterial strains from animals to humans, these findings may be of great importance. By potentially affecting the emergence of resistant strains, copper can indirectly contribute to the reduced effects of antibiotics. Several examples of links between Cu ions and bacterial resistance are described such as: Cu influence on the promotor gene in Gram-negative bacteria responsible for the permeability of the cell membrane, influence on a regulatory gene linked with drug efflux pump, horizontal gene transfer, expression of the mycobacteria repressor gene, reduced uptake, extracellular and/or intracellular sequestration, metabolic bypass, or chemical modification [23,29,95,96,97]. Mechanisms of defense mediated through ATPase export pumps are nowadays marked as an adaptation behavior and are found mostly in microorganisms living in Cu-rich environments [31]. ATPase export pumps prevent Cu accumulation in the cytoplasm by using the energy from ATP hydrolysis to pump Cu^1+^ across the plasma membrane in Gram-positive bacteria or across the inner membrane to periplasmic space in Gram-negative bacteria [98]. Many Gram-negative bacteria synthesize multicopper oxidases, enzymes responsible for the transformation of Cu^+^ into the less toxic Cu^2+^ form [31]. Bacterial metallothioneins play a role in Cu detoxification by metal sequestration [99]. Genetic linkage of Cu and antibiotic resistance genes in bacteria is described in aquatic, livestock, and human environments [69]. Increased expression of copper tolerance genes is achieved through the impact of high Cu concentration on the stimulation of numerous transcription factors or on the inhibition of transcriptional repressor genes [98].

## 7. Future Perspectives

Based on the discussed Cu–antibiotic interaction findings, several steps should be implemented in order to achieve a benefit in clinical settings, adding the warnings in the guideline recommendations for antibiotics use in patients with altered Cu levels. Conducting in vitro studies in physiological pH settings and well-designed randomized placebo-controlled trials in patients with Cu elevation levels would significantly improve the present knowledge regarding Cu–antibiotic complexes formation and stability as well as their efficacy. A step further would be to investigate the full spectrum of activity of Cu-dependent compounds with special attention toward their possible toxic effects and influence on inflammatory parameters.

## 8. Conclusions

Given the development of new antibiotics is already slower compared to the global needs, the impact of Cu on antibiotic activity should be carefully examined. Although the antimicrobial role of copper is well known, the exact mechanisms are still questionable. So far, in vitro-described degradation or lower antimicrobial activity in the presence of Cu and antibiotics such as amoxicillin, ampicillin, meropenem, several cephalosporins, and neomycin suggest caution when using these antibiotics in patients with conditions that are linked to the elevated Cu concentration. On the other hand, the possible role of copper-dependent compounds in preventing and reversing antimicrobial resistance can be of clinical value. Due to the lack of in vitro studies in physiological conditions and translation into in vivo studies, many questions regarding Cu–antibiotics interactions still remain open: chemical groups involved in interactions, pharmacokinetic properties of complexes formed, stability, and possible changes in antimicrobial activity. We believe that the missing pieces of the puzzle regarding the roles of copper and its complexes in antimicrobial therapy in clinical settings will be provided by multidisciplinary approaches in the future.

## Figures and Tables

**Table 1:** Examples of Cu-dependent compounds and microorganisms they are active against.

Cu-Dependent Compounds	Microorganisms
N4-methyl-thiosemicarbazones	Methicillin-resistant *Staphylococcus aureus* (MRSA), *M. tuberculosis*, *N. gonorrhoeae*, *S. pneumoniae*, *H. influenza* [32,33]
Disulfiram	*M. tuberculosis* [34]
Thiocarlide	MRSA [33]
8-hydroxyquinoline	*M. tuberculosis*, *C. Neoformans*, *L. monocytogenes* [1,35]
1,10,phenanthroline	*A. baumannii*, *P. aeruginosa* [36]
Neocuproine	MRSA, *M. gallisepticum*, *P. denitrificans* [1,33]
*P yrazolopyrimidinone*	*S. aureus* [37]
*P yrithione*	*K. pneumoniae* [38]

**Table 2:** Antimicrobial activity of selected cephalosporins in the presence of Cu^2+^ compared to antibiotic alone.

	Antimicrobial Activity of Selected Antibiotics in the Presence of Copper						
Antibiotic	*E. coli*	*S. aureus*	*K. pneumoniae*	*P. mirabilis*	*S. enteriditis*	*S. sonnei*	*B. subtilis*
Cephalexin	↑ [5,57,58]	↑ [57,58] No changes [5]	No changes [58]	↑ [58]	↑ [58]		
Cephadroxil	No changes [62]						No changes [62]
Cefradine	↑ [57,59]	↓ [57,59]		↓ [59]	↓ [59]	↓ [59]	
Cefazolin	↑ [57] No changes [60]	No changes [57,60]	No changes [60]		No changes [60]		No changes [60]
Cefaclor	↑ [5]	No changes [5]					
Ceftriaxone	↓ [5,57,60,61]	↓ [5,57,60,61]	↓ [60]		↓ [60]		↓ [60] ↑ [57]
Ceftazidime	No changes [5]	↓ [5]					
Cefepime	↑ [60]	↑ [60]	↓ [60]				No changes [60]

Information on antimicrobial activity in Table 1 is based on the observed MIC changes (↑—increased effect; ↓—decreased effect).

## Data Availability

Not applicable.

## References

[1] Dalecki A.G., Crawford C.L., Wolschendorf F. (2017). Copper and Antibiotics: Discovery, Modes of Action, and Opportunities for Medicinal Applications. Adv. Microb. Physiol..

[2] Lewis K. (2013). Platforms for antibiotic discovery. Nat. Rev. Drug Discov..

[3] Antimicrobial Resistance Collaborators (2022). Global burden of bacterial antimicrobial resistance in 2019: A systematic analysis. Lancet.

[4] Crawford C.L., Dalecki A.G., Perez M.D., Schaaf K., Wolschendorf F., Kutsch O. (2020). A copper-dependent compound restores ampicillin sensitivity in multidrug-resistant Staphylococcus aureus. Sci. Rep..

[5] Božić B., Korać J., Stanković D.M., Stanić M., Romanović M., Pristov J.B., Spasić S., Popović-Bijelić A., Spasojević I., Bajčetić M. (2018). Coordination and redox interactions of β-lactam antibiotics with Cu^2+^ in physiological settings and the impact on antibacterial activity. Free Radic. Biol. Med..

[6] Ince A.T., Kayadibi H., Soylu A., Ovunç O., Gültepe M., Toros A.B., Yaşar B., Kendir T., Abut E. (2008). Serum copper, ceruloplasmin and 24-h urine copper evaluations in celiac patients. Dig. Dis. Sci..

[7] Iakovidis I., Delimaris I., Piperakis S.M. (2011). Copper and Its Complexes in Medicine: A Biochemical Approach. Mol. Biol. Int..

[8] Twomey P.J., Reynolds T.M., Wierzbicki A.S., Viljoen A. (2008). The relationship between serum copper and ceruloplasmin in routine clinical practice. Int. J. Clin. Pract..

[9] Schulpis K.H., Karakonstantakis T., Gavrili S., Costalos C., Romac E., Papassotiriou I. (2004). Serum copper is decreased in premature newborns and increased in newborns with hemolytic jaundice. Clin. Chem..

[10] Yoshikawa S., Muramoto K., Shinzawa-Itoh K. (2012). Reaction mechanism of mammalian mitochondrial cytochrome c oxidase. Adv. Exp. Med. Biol..

[11] Smith-Mungo L.I., Kagan H.M. (1998). Lysyl oxidase: Properties, regulation and multiple functions in biology. Matrix Biol..

[12] Valentine J.S., Doucette P.A., Zittin Potter S. (2005). Copper-zinc superoxide dismutase and amyotrophic lateral sclerosis. Annu. Rev. Biochem..

[13] Shepard E.M., Dooley D.M. (2015). Inhibition and oxygen activation in copper amine oxidases. Acc. Chem. Res..

[14] Akyilmaz E., Yorganci E., Asav E. (2010). Do copper ions activate tyrosinase enzyme? A biosensor model for the solution. Bioelectrochemistry.

[15] Lowe J., Taveira-da-Silva R., Hilário-Souza E. (2017). Dissecting copper homeostasis in diabetes mellitus. IUBMB Life.

[16] Moraes M.L., Ramalho D.M., Delogo K.N., Miranda P.F., Mesquita E.D., de Melo Guedes de Oliveira H.M., Netto A.R., Dos Anjos M.J., Kritski A.L., de Oliveira M.M. (2014). Association of serum levels of iron, copper, and zink, and inflammatory markers with bacteriological sputum conversion during tuberculosis treatment. Biol. Trace Elem. Res..

[17] Bost M., Houdart S., Oberli M., Kalonji E., Huneau J.F., Margaritis I. (2016). Dietary copper and human health: Current evidence and unresolved issues. J. Trace Elem. Med. Biol..

[18] Georgieff M.K. (2007). Nutrition and the developing brain: Nutrient priorities and measurement. Am. J. Clin. Nutr..

[19] Pourahmad J., O’Brien P.J. (2000). A comparison of hepatocyte cytotoxic mechanisms for Cu^2+^ and Cd^2+^. Toxicology.

[20] Bonda D.J., Liu G., Men P., Perry G., Smith M.A., Zhu X. (2012). Nanoparticle Delivery of Transition-Metal Chelators to the Brain: Oxidative Stress will Never See it Coming! CNS Neurol Disord Drug Targets. CNS Neurol. Disord. Drug Targets.

[21] Viktorinova A. (2017). Current insights on the role of iron and copper dyshomeostasis in the pathogenesis of bilirubin neurotoxicity. Life Sci..

[22] Grass G., Rensing C., Solioz M. (2011). Metallic copper as an antimicrobial surface. Appl. Environ. Microbiol..

[23] Lemire J.A., Harrison J.J., Turner R.J. (2013). Antimicrobial activity of metals: Mechanisms, molecular targets and applications. Nat. Rev. Microbiol..

[24] Mozafari M.R., Sarabanou T., Karamouzian F.M., Fatemeh M., Babak R., Bikash B. (2021). Antimicrobial Applications of Nanoliposome Encapsulated Silver Nanoparticles: A Potential Strategy to Overcome Bacterial Resistance. Curr. Nanosci..

[25] Usman M.S., El Zowalaty M.E., Shameli K., Zainuddin N., Salama M., Ibrahim N.A. (2013). Synthesis, characterization, and antimicrobial properties of copper nanoparticles. Int. J. Nanomed..

[26] Gaetke L.M., Chow C.K. (2003). Copper toxicity, oxidative stress, and antioxidant nutrients. Toxicology.

[27] Teitzel G.M., Geddie A., De Long S.K., Kirisits M.J., Whiteley M., Parsek M.R. (2006). Survival and growth in the presence of elevated copper: Transcriptional profiling of copper-stressed Pseudomonas aeruginosa. J. Bacteriol..

[28] Buettner G.R. (1993). The pecking order of free radicals and antioxidants: Lipid peroxidation, alpha-tocopherol, and ascorbate. Arch. Biochem. Biophys..

[29] Harrison J.J., Tremaroli V., Stan M.A., Chan C.S., Vacchi-Suzzi C., Heyne B.J., Parsek M.R., Ceri H., Turner R.J. (2009). Chromosomal antioxidant genes have metal ion-specific roles as determinants of bacterial metal tolerance. Environ. Microbiol..

[30] Macomber L., Imlay J.A. (2009). The iron-sulfur clusters of dehydratases are primary intracellular targets of copper toxicity. Proc. Natl. Acad. Sci. USA.

[31] Vincent M., Duval R.E., Hartemann P., Engels-Deutsch M. (2018). Contact killing and antimicrobial properties of copper. J. Appl. Microbiol..

[32] Djoko K.Y., Goytia M.M., Donnelly P.S., Schembri M.A., Shafer W.M., McEwan A.G. (2015). Copper(II)-Bis(Thiosemicarbazonato) Complexes as Antibacterial Agents: Insights into Their Mode of Action and Potential as Therapeutics. Antimicrob. Agents Chemother..

[33] Haeili M., Moore C., Davis C.J., Cochran J.B., Shah S., Shrestha T.B., Zhang Y., Bossmann S.H., Benjamin W.H., Kutsch O. (2014). Copper complexation screen reveals compounds with potent antibiotic properties against methicillin-resistant Staphylococcus aureus. Antimicrob. Agents Chemother..

[34] Dalecki A.G., Haeili M., Shah S., Speer A., Niederweis M., Kutsch O., Wolschendorf F. (2015). Disulfiram and Copper Ions Kill Mycobacterium tuberculosis in a Synergistic Manner. Antimicrob. Agents Chemother..

[35] Shah S., Dalecki A.G., Malalasekera A.P., Crawford C.L., Michalek S.M., Kutsch O., Sun J., Bossmann S.H., Wolschendorf F. (2016). 8-Hydroxyquinolines Are Boosting Agents of Copper-Related Toxicity in Mycobacterium tuberculosis. Antimicrob. Agents Chemother..

[36] Ventura R.F., Galdino A.C.M., Viganor L., Schuenck R.P., Devereux M., McCann M., Santos A.L.S., Nunes A.P.F. (2020). Antimicrobial action of 1,10-phenanthroline-based compounds on carbapenemase-producing Acinetobacter baumannii clinical strains: Efficacy against planktonic- and biofilm-growing cells. Braz. J. Microbiol..

[37] Crawford C.L., Dalecki A.G., Naramore W.T., Hoff J., Hargett A.A., Renfrow M.B., Zhang M., Kalubowilage M., Bossmann S.H., Queern S.L. (2019). Pyrazolopyrimidinones, a novel class of copper-dependent bacterial antibiotics against multi-drug resistant S. aureus. Metallomics.

[38] Chiem K., Fuentes B.A., Lin D.L., Tran T., Jackson A., Ramirez M.S., Tolmasky M.E. (2015). Inhibition of aminoglycoside 6′-N-acetyltransferase type Ib-mediated amikacin resistance in Klebsiella pneumoniae by zinc and copper pyrithione. Antimicrob. Agents Chemother..

[39] Djoko K.Y., Paterson B.M., Donnelly P.S., McEwan A.G. (2014). Antimicrobial effects of copper(II) bis(thiosemicarbazonato) complexes provide new insight into their biochemical mode of action. Metallomics.

[40] Reeder N.L., Kaplan J., Xu J., Youngquist R.S., Wallace J., Hu P., Juhlin K.D., Schwartz J.R., Grant R.A., Fieno A. (2011). Zinc pyrithione inhibits yeast growth through copper influx and inactivation of iron-sulfur proteins. Antimicrob. Agents Chemother..

[41] Ng N.S., Leverett P., Hibbs D.E., Yang Q., Bulanadi J.C., Wu M.J., Aldrich-Wright J.R. (2013). The antimicrobial properties of some copper(II) and platinum(II) 1,10-phenanthroline complexes. Dalton Trans..

[42] Galdino A.C.M., Viganor L., Pereira M.M., Devereux M., McCann M., Branquinha M.H., Molphy Z., O’Carroll S., Bain C., Menounou G. (2022). Copper(II) and silver(I)-1,10-phenanthroline-5,6-dione complexes interact with double-stranded DNA: Further evidence of their apparent multi-modal activity towards Pseudomonas aeruginosa. J. Biol. Inorg. Chem..

[43] Deegan C., Coyle B., McCann M., Devereux M., Egan D.A. (2006). In vitro anti-tumour effect of 1,10-phenanthroline-5,6-dione (phendione), [Cu(phendione)_3_](ClO_4_)_2_.4H_2_O and [Ag(phendione)_2_]ClO_4_ using human epithelial cell lines. Chem. Biol. Interact..

[44] Baym M., Stone L.K., Kishony R. (2016). Multidrug evolutionary strategies to reverse antibiotic resistance. Science.

[45] Lima L.M., Silva B.N.M.D., Barbosa G., Barreiro E.J. (2020). β-lactam antibiotics: An overview from a medicinal chemistry perspective. Eur. J. Med. Chem..

[46] De Rosa M., Verdino A., Soriente A., Marabotti A. (2021). The Odd Couple(s): An Overview of Beta-Lactam Antibiotics Bearing More Than One Pharmacophoric Group. Int. J. Mol. Sci..

[47] Miller E.L. (2002). The penicillins: A review and update. J. Midwifery Womens Health..

[48] Kong K.F., Schneper L., Mathee K. (2010). Beta-lactam antibiotics: From antibiosis to resistance and bacteriology. APMIS.

[49] Abraham E.P., Newton G.G.F. (1961). The structure of cephalosporin C. Biochem. J..

[50] Möhler J.S., Kolmar T., Synnatschke K., Hergert M., Wilson L.A., Ramu S., Elliott A.G., Blaskovich M.A., Sidjabat H.E., Paterson D.L. (2017). Enhancement of antibiotic-activity through complexation with metal ions—Combined ITC, NMR, enzymatic and biological studies. J. Inorg. Biochem..

[51] Cressman W.A., Sugita E.T., Doluisio J.T., Niebergall P.J. (1966). Complexation of penicillins and penicilloic acids by cupric ion. J. Pharm. Pharmacol..

[52] Niebergall P.J., Hussar D.A., Cressman W.A., Sugita E.T., Doluisio J.T. (1966). Metal binding tendencies of various antibiotics. J. Pharm. Pharmacol..

[53] Lapshin S.V., Alekseev V.G. (2009). Copper(II) complexation with ampicillin, amoxicillin, and cephalexin. Russ. J. Inorg. Chem..

[54] Guo Y., Tsang D.C.W., Zhang X., Yang X. (2018). Cu(II)-catalyzed degradation of ampicillin: Effect of pH and dissolved oxygen. Environ. Sci. Pollut. Res. Int..

[55] Chohan Z.H., Supuran C.T., Scozzafava A. (2004). Metalloantibiotics: Synthesis and antibacterial activity of cobalt(II), copper(II), nickel(II) and zinc(II) complexes of kefzol. J. Enzyme Inhib. Med. Chem..

[56] El-Gamel N.E.A. (2010). Metal chelates of ampicillin versus amoxicillin: Synthesis, structural investigation, and biological studies. J. Coord. Chem..

[57] Auda S.H., Mrestani Y., Fetouh M.I., Neubert R.H. (2008). Characterization and activity of cephalosporin metal complexes. Pharmazie.

[58] Anacona J.R., Rodrigues I. (2004). Synthesis and antibacterial activity of cephalexin metal complexes. J. Coord. Chem..

[59] Anacona J.R., Acosta F. (2005). Synthesis and antibacterial activity of cephradine metal complexes. J. Coord. Chem..

[60] Anacona J.R., Osorio I. (2008). Synthesis and antibacterial activity of copper(II) complexes with sulphathiazole and cephalosporin ligands. Transit. Met. Chem..

[61] Ali A.E. (2011). Synthesis, spectral, thermal and antimicrobial studies of some new tri metallic biologically active ceftriaxone complexes. Spectrochim. Acta A Mol. Biomol. Spectrosc..

[62] Auda S.H., Knütter I., Bretschneider B., Brandsch M., Mrestani Y., Große C., Neubert R.H. (2009). Effect of Different Metal Ions on the Biological Properties of Cefadroxil. Pharmaceuticals.

[63] Singh B.K., Bhojak N., Mishra P., Garg B.S. (2008). Copper(II) complexes with bioactive carboxyamide: Synthesis, characterization and biological activity. Spectrochim. Acta A Mol. Biomol. Spectrosc..

[64] Chohan Z.H., Pervez H., Khan K.M., Rauf A., Supuran C.T. (2004). Binding of Transition Metal Ions [Cobalt, Copper, Nickel and Zinc] with Furanyl-, Thiophenyl-, Pyrrolyl-, Salicylyland Pyridyl-Derived Cephalexins as Potent Antibacterial Agents. J. Enzyme Inhib. Med. Chem..

[65] Djoko K.Y., Achard M.E.S., Phan M.D., Lo A.W., Miraula M., Prombhul S., Hancock S.J., Peters K.M., Sidjabat H.E., Harris P.N. (2018). Copper Ions and Coordination Complexes as Novel Carbapenem Adjuvants. Antimicrob. Agents Chemother..

[66] Aurilio C., Sansone P., Barbarisi M., Pota V., Giaccari L.G., Coppolino F., Barbarisi A., Passavanti M.B., Pace M.C. (2022). Mechanisms of Action of Carbapenem Resistance. Antibiotics.

[67] Zhao Y., Tan Y., Guo Y., Gu X., Wang X., Zhang Y. (2013). Interactions of tetracycline with Cd (II), Cu (II) and Pb (II) and their cosorption behavior in soils. Environ. Pollut..

[68] Chopra I., Roberts M. (2001). Tetracycline antibiotics: Mode of action, applications, molecular biology, and epidemiology of bacterial resistance. Microbiol. Mol. Biol. Rev..

[69] Poole K. (2017). At the Nexus of Antibiotics and Metals: The Impact of Cu and Zn on Antibiotic Activity and Resistance. Trends Microbiol..

[70] Tong F., Zhao Y., Gu X., Gu C., Lee C.C. (2015). Joint toxicity of tetracycline with copper(II) and cadmium(II) to Vibrio fischeri: Effect of complexation reaction. Ecotoxicology.

[71] Feio M.J., Sousa I., Ferreira M., Cunha-Silva L., Saraiva R.G., Queirós C., Alexandre J.G., Claro V., Mendes A., Ortiz R. (2014). Fluoroquinolone-metal complexes: A route to counteract bacterial resistance?. J. Inorg. Biochem..

[72] Sousa I., Claro V., Pereira J.L., Amaral A.L., Cunha-Silva L., de Castro B., Feio M.J., Pereira E., Gameiro P. (2012). Synthesis, characterization and antibacterial studies of a copper(II) levofloxacin ternary complex. J. Inorg. Biochem..

[73] Saraiva R., Lopes S., Ferreira M., Novais F., Pereira E., Feio M.J., Gameiro P. (2010). Solution and biological behaviour of enrofloxacin metalloantibiotics: A route to counteract bacterial resistance?. J. Inorg. Biochem..

[74] Fernandes P., Sousa I., Cunha-Silva L., Ferreira M., de Castro B., Pereira E.F., Feio M.J., Gameiro P. (2014). Synthesis, characterization and antibacterial studies of a copper(II) lomefloxacin ternary complex. J. Inorg. Biochem..

[75] Efthimiadou E.K., Katsarou M.E., Karaliota A., Psomas G. (2008). Copper(II) complexes with sparfloxacin and nitrogen-donor heterocyclic ligands: Structure-activity relationship. J. Inorg. Biochem..

[76] Patel M.N., PArmar P.A., Gandhi D.S. (2010). Square pyramidal copper(II) complexes with forth generation fluoroquinolone and neutral bidentate ligand: Structure, antibacterial, SOD mimic and DNA-interaction studies. Bioorg. Med. Chem..

[77] Kotra L.P., Haddad J., Mobashery S. (2000). Aminoglycosides: Perspectives on mechanisms of action and resistance and strategies to counter resistance. Antimicrob. Agents Chemother..

[78] Lesniak W., Harris W.R., Kravitz J.Y., Schacht J., Pecoraro V.L. (2003). Solution chemistry of copper(II)-gentamicin complexes: Relevance to metal-related aminoglycoside toxicity. Inorg. Chem..

[79] Toth M., Frase H., Antunes N.T., Smith C.A., Vakulenko S.B. (2010). Crystal structure and kinetic mechanism of aminoglycoside phosphotransferase-2″-IVa. Protein Sci..

[80] Szczepanik W., Kaczmarek P., Jezowska-Bojczuk M. (2004). Oxidative activity of copper(II) complexes with aminoglycoside antibiotics as implication to the toxicity of these drugs. Bioinorg. Chem. Appl..

[81] Song B.B., Sha S.H., Schacht J. (1998). Iron chelators protect from aminoglycoside-induced cochleo- and vestibulo-toxicity. Free Radic. Biol. Med..

[82] Szczepanik W., Dworniczek E., Ciesiołka J., Wrzesiński J., Skala J., Jezowska-Bojczuk M. (2003). In vitro oxidative activity of cupric complexes of kanamycin A in comparison to in vivo bactericidal efficacy. J. Inorg. Biochem..

[83] Abu-el-wafa S.M., El-ries M.A., Abou-attia F.M., Issa R.M. (1989). Coordination chemical studies of some polymeric transition metal complexes with neomycin and their biological activity uses. Indirect determination of neomycin by atomic absorption spectroscopy (AAS). Anal. Lett..

[84] Miljkovic V., Arsic B., Bojanic Z., Nikolic G., Nikolic L., Kalicanin B., Savic V. (2014). Interactions of metronidazole with other medicines: A brief review. Pharmazie.

[85] Palmer J.H., Wub J.S., Upmacis R.K. (2015). Coordination of metronidazole to Cu(II): Structural characterization of a mononuclear square-planar compound. J. Mol. Struct..

[86] Galván-Tejada N., Bernès S., Castillo-Blum S.E., Nöth H., Vicente R., Barba-Behrens N. (2002). Supramolecular structures of metronidazole and its copper(II), cobalt(II) and zinc(II) coordination compounds. J. Inorg. Biochem..

[87] Rafique B., Shafique K., Hamid S., Kalsoom S., Hashim M., Mirza B., Jafri L., Iqbal M. (2022). Novel copper complexes of metronidazole and metronidazole benzoate: Synthesis, characterization, biological and computational studies. J. Biomol. Struct. Dyn..

[88] Wijesekara P.N.K., Kumbukgolla W.W., Jayaweera J.A.A.S., Rawat D. (2017). Review on Usage of Vancomycin in Livestock and Humans: Maintaining Its Efficacy, Prevention of Resistance and Alternative Therapy. Vet. Sci..

[89] Swiatek M., Valensin D., Migliorini C., Gaggelli E., Valensin G., Jezowska-Bojczuk M. (2005). Unusual binding ability of vancomycin towards Cu^2+^ ions. Dalton Trans..

[90] Hanson J.C., Camerman N., Camerman A. (1981). Structure of a copper-isoniazid complex. J. Med. Chem..

[91] Sakurai H., Shimomura S., Ishizu K. (1980). Green and purple copper (II)-chloramphenicol complexes in methanol: Evidence for the coordination of deprotonated amide nitrogen. J. Antibiot..

[92] Jezowska-Bojczuk M., Lesniak W., Szczepanik W., Gatner K., Jezierski A., Smoluch M., Bal W. (2001). Copper(II)-lincomycin: Complexation pattern and oxidative activity. J. Inorg. Biochem..

[93] Hamdan I.I. (2003). Comparative in vitro investigations of the interaction between some macrolides and Cu(II), Zn(II) and Fe(II). Pharmazie.

[94] Anedda E., Farrell M.L., Morris D., Burgess C.M. (2023). Evaluating the impact of heavy metals on antimicrobial resistance in the primary food production environment: A scoping review. Environ. Pollut..

[95] Yamamoto K., Ishihama A. (2006). Characterization of copper-inducible promoters regulated by CpxA/CpxR in Escherichia coli. Biosci. Biotechnol. Biochem..

[96] Song Z., Zuo L., Li C., Tian Y., Wang H. (2021). Copper Ions Facilitate the Conjugative Transfer of SXT/R391 Integrative and Conjugative Element Across Bacterial Genera. Front. Microbiol..

[97] Rao M., Liu H., Yang M., Zhao C., He Z.G. (2012). A copper-responsive global repressor regulates expression of diverse membrane-associated transporters and bacterial drug resistance in Mycobacteria. J. Biol. Chem..

[98] Ladomersky E., Petris M.J. (2015). Copper tolerance and virulence in bacteria. Metallomics.

[99] Chatterjee S., Kumari S., Rath S., Priyadarshanee M., Das S. (2020). Diversity, structure and regulation of microbial metallothionein: Metal resistance and possible applications in sequestration of toxic metals. Metallomics.

